# Telephone-delivered health behaviour change support for people with a mental health condition: the coaches’ perspective

**DOI:** 10.1186/s12913-021-07126-4

**Published:** 2021-10-21

**Authors:** Tegan Bradley, Vibeke Hansen, Paula Wye, Elizabeth Campbell, Kate Bartlem, Kate Reid, Jenny Bowman

**Affiliations:** 1 University of Newcastle, University Drive, Callaghan, NSW 2308 Australia; 2 Hunter Medical Research Institute, Lot 1, Kookaburra Cct, New Lambton Heights, NSW 2305 Australia; 3 Southern Cross University, Hogbin Dr, Coffs Harbour, NSW 2450 Australia; 4 Hunter New England Population Health, Locked Bag 10, Wallsend, NSW 2287 Australia; 5 NSW Office of Preventive Health, Liverpool Hospital, Don Everett Building, Locked Bag 7103, Liverpool, NSW BC1871 Australia

**Keywords:** Preventive health services, Mental health, Chronic disease, Physical activity, Weight management, Health care quality, Qualitative research, Health coaching

## Abstract

**Background:**

People with a mental health condition experience a greater prevalence of chronic disease and reduced life expectancy compared to the general population. Modifiable health risk behaviours, such as physical inactivity and poor nutrition are major contributing factors. Population-level health coaching delivering behavioural change support via telephone for healthy eating, physical activity, and weight management is an opportunity utilised by this group to support improvement in healthy lifestyle behaviours. Health coaches offer a valuable perspective into the provision of services to this high-risk group. This study aims to qualitatively explore coaches’ experiences in providing support to these participants, consider factors which may contribute to engagement and outcomes; and potentially inform future service improvement.

**Method:**

A qualitative study design was employed involving semi-structured telephone interviews with six coaches employed in a telephone-based behaviour change support service in New South Wales, Australia, between April and July 2019. Interview data was analysed using an inductive thematic analysis.

**Results:**

Coaches believed that the service was of benefit to people with a mental health condition, however making changes to health risk behaviours was potentially more difficult for this group of service users. Coaches indicated that in supporting this group there was a greater focus on building confidence and readiness to change. They noted that improvement in mental health as a result of physical health changes was an additional ‘measure of success’ of particular relevance. Coaches expressed a desire to receive more mental health training to better deliver coaching to participants with a mental health condition. Program variables such as limited call length were posed as possible barriers to care.

**Conclusion:**

Further training and additional support for coaches, in additon to considering variations to aspects of service delivery may assist in improving engagement and outcomes for participants with mental health conditions. Examining mental health consumers’ experiences when engaging with telephone coaching services would be an important area to address in further research.

Cumulative evidence has confirmed an elevated risk of chronic physical health conditions for people with any mental health condition when compared to the general population [1, 2], leading to a substantially reduced life expectancy globally of up to 32 years [3–5]. Such conditions, including cardiovascular disease and diabetes, have been linked to modifiable health risk behaviours including smoking, poor nutrition and physical inactivity. These risk factors have a higher prevalence among people with a mental health condition, with global estimates that up to 70% of people with a mental health condition are not meeting recommended physical activity guidelines, compared to 25% of the general population [6–8]. Supporting people with a mental health condition to make changes to their health risk behaviours is an identified policy priority in Australia and internationally [9, 10].

Telephone services that provide behaviour change support have been found to be effective for the general population. Systematic reviews of health interventions delivered via telephone have found this method of delivery effective in improving health behaviours such as smoking cessation [11], increased physical activity and healthy eating [12],and cost-effective [8] in the general population. When provided as population-level prevention, telephone-delivered services can provide highly accessible expert coaching to improve health risk behaviours, exemplified internationally by Quitlines for smoking cessation [11]. Similarly, The NSW Get Healthy Information and Coaching Service®(GHS) provides free health coaching advice for Australian adults residing in the states of New South Wales, South Australia and Queensland [13]; with those who complete a 6-month coaching program reported to make sustained improvements in healthy eating (fruit and vegetable intake), physical activity and achieving or maintaining a healthy weight [13]. Evidence suggests that the advantages of telephone-delivered services include provision of an easy referral pathway for health professionals [14], and the element of social support provided by coaches who deliver the telephone service; the latter improving the likelihood of successful behaviour change [15].

People with a mental health condition have been reported to be well-represented among the participants of population-level telephone coaching services. For example, approximately half of callers to three state Quitlines within the United States of America had an identified mental health condition [16, 17], while a third of callers to an Australian state Quitline [18] and a quarter of service users accessing the GHS indicated having a mental health condition [19]. Telephone services may be particularly advantageous for this high risk population, where features such as flexibility in call schedules may assist in overcoming reported barriers to participating in face to face health interventions leading to an inability to attend sessions [20–22], conflicting appointments [23–25] and transport issues [22, 26, 27]. Research on behaviour change outcomes to date has been limited to Quitlines and indicates that although Quitlines are effective for callers with a mental health condition, these callers achieve poorer cessation outcomes after making contact with the service compared to those without a mental health condition [16–18, 28–30].

Input from a number of stakeholders, including consumers and service providers, is critical in evaluating behaviour change services [31]. Coaches who deliver programs provide a valuable and unique perspective on factors relating to engagement with, and outcomes achieved from health behaviour change support services by mental health participants. Research has identified factors that may represent particular barriers or challenges to health behaviour change for people with a mental health condition: medication side effects [32], lower confidence in health behaviour change [19] and a sense of powerlessness in improving physical health [33]. Greater understanding of how population-level telephone coaching services and coaches perceive and respond to such factors may improve service delivery and outcomes for this important participant group.

No literature was found which reports the experiences of health coaches in providing telephone support for modifiable health risk behaviours to people with a mental health condition. The present study aims to qualitatively explore the views and experiences of the GHS coaches who provide support to improve physical activity, diet and weight. More specifically, it will explore experiences in providing a telephone-based health behaviour change coaching program to participants who indicated having a mental health condition, coaches’ perceptions of factors that may impact program engagement and outcomes for this group of participants, and draw on those observations to identify possible areas for service improvement.

## Methods

### Design and setting

Semi-structured interviews were conducted with health coaches within a free, government-funded telephone-based behaviour change support service, the GHS. The service is a customer-focused, clinically integrated primary and secondary prevention service available to residents 16 years and above in New South Wales and Queensland, or 18 years and above in South Australia. Individuals can self-refer to the service via the website or by calling directly, or they can be referred by a health professional. The service offers healthy lifestyle coaching and education programs to address multiple lifestyle-related risk factors including support for healthy eating, improving physical activity, achieving or maintaining a healthy weight, and alcohol reduction [13]. Participants enrolled in a coaching program are allocated a university-qualified health coach. Coaches receive training in motivational interviewing, focusing on working effectively with patients to promote: health literacy; shared and fully-informed decision-making; and evidence-based, goal-oriented treatment, self-management of health behaviours [34]. Health coaches provide up to 13 proactive calls over a 6 month period, with each coaching call lasting approximately 15–20 min. Coaching calls are provided on a tapered schedule, with a higher intensity of calls occurring in the first 12 weeks of the program to promote initiation of behaviour change, and less frequent calls during the latter 14 weeks to promote maintenance and prevent relapse. Coaching calls are scheduled between the participant and health coach in a personalised and person-centred way, creating flexibility for participants to have more or less frequent calls.

Interviews were deemed the most appropriate approach to data collection, over other approaches including focus groups, due to both the logistical difficulty of having multiple coaches available at the same time, as well as the likelihood that participants may choose to withhold information in the presence of other service employees as topics addressed included sensitive material around experiences and opinions [35, 36]. The study was approved by the University of Newcastle’s Human Research Ethics Committee (H-2018-0388).

### Participants and recruitment

An email invitation to participate was distributed by the service manager to all health coaches employed in April, 2019. The email included a participant information sheet outlining the study purpose and aims, as well as a link for potential participants to ‘opt in’ and complete a brief, anonymous online survey, with the option to provide contact details if participants were willing to complete an interview during a paid shift with the service.

The survey collected the following information regarding the coaches and their experience in providing behaviour change support: gender; year of birth; Aboriginal and/or Torres Strait Islander identification; professional background; previous experience in providing health behaviour change support prior to employment with this service; previous experience providing care to people with a mental health condition; previous experience providing care to people living in rural/remote areas; length of time employed by the service; and capacity to provide the coaching in a language other than English; the number of participants with whom they had started a coaching program and the number of participants who had completed a coaching program with them. To enhance the accuracy of information for the last two variables, this information was generated by the service and provided to health coaches prior to the email invitation being distributed.

### Interview procedure

Coaches willing to participate were contacted by the research team to arrange a telephone or zoom interview with an experienced qualitative researcher (VH). Health coaches completed interviews during scheduled shifts with the service, and verbal consent to participate was gained prior to interview commencement. The interviewer (VH) was provided minimal information aside from the interview topic guide (see below) prior to the interviews to improve confirmability through minimisation of investigator bias. Coaches were informed that the purpose of the interview was to understand their experiences of delivering a behaviour change service to participants with a mental health condition as part of the coaching role. Coaches were informed that a copy of their transcribed interview would be made available upon request.

A semi-structured interview guide was used to guide discussion. Questions were centred around coaches’ experience in providing behaviour change support to people with a mental health condition as a part of the coaching role; factors that may impact the effectiveness of the service for this group of service users, including both service-participant, coach and organisational factors; and suggestions for any changes to the delivery of the service and outcomes for callers with a mental health condition. Prompts were used in the interview to facilitate elaboration on aspects pertaining to experiences of the coach, potential factors that may impact behaviour change for people with a mental health condition*,* as well as any recommendations for improving outcomes for this group of service users*.*

### Analysis

Interview audio recordings were transcribed verbatim and NVivo 11 was used to assist with organisational aspects of the analysis. An inductive thematic analysis was undertaken by authors TB, VH & PW, who independently generated codes from the first transcript, engaged in discussion around discrepancies and reached consensus on a draft coding hierarchy. This hierarchy was then further developed and refined during coding of the remaining five transcripts, which were coded by author TB. Once all transcripts were coded, further discussions between authors TB, VH, PW, JB and EC led to the generation of a thematic structure which was further refined during repeated engagement with the data, to ensure that the themes generated resonated with the story of the complete data set.

## Results

Fourteen of 19 coaches employed at the time of email distribution completed the online survey (74%), with results shown in Table 1. Six coaches indicated they were willing to participate in an interview and provided their contact details.

**Table 1:** Health coach survey responses (*n* = 14)

Demographic	Response
Experience providing behaviour change support (months)^a^	Mean: 14 months Range: 0–38 months Median: 16 months
Length of employment with the service	Mean: 22 months Range: 1 month to 7 years Median: 16 months
Professional background^b^	Dietetics (*n* = 10, 71%) Exercise Physiology (*n* = 4, 29%) Nursing (*n* = 1, 7%)
Previous provision of care to people in rural/remote areas^a^	None (*n* = 11, 79%) Less than 12 months (*n* = 3, 21%)
Previous provision of care to people with a mental health condition^a^	None (*n* = 6, 43%) Less than 12 months (*n* = 6, 43%) More than 12 months (*n* = 2, 14%)
Can provide support in a language other than English	No (*n* = 12, 86%) Yes (*n* = 2, 14%)
Number of participants whom had started a coaching program	< 50 (*n* = 3, 21%) 50–100 (*n* = 1, 7%) 200+ (*n* = 10, 71%)
Number of participants whom had completed a coaching program	< 50 (*n* = 7, 50%) 50–100 (*n* = 5, 36%) 100+ (*n* = 2, 14%)
Gender^c^	Female (*n* = 14, 100%)
Aboriginal and/or Torres Strait Islander	No (*n* = 14, 100%)

^a^Prior to employment with the service

^b^Multiple options could be presented

^c^Coaches employed at the time of the study included both males and females, though predominantly female (GHS personal communication)

Six coaches completed a telephone interview between May and July 2019, all of whom were female, with a mean age of 26 years. Interview duration ranged from 16 to 49 min (mean 27 min). Coaches who participated in the interviews had, on average: worked with the service for 18 months, and 10 months experience in providing health behaviour change advice prior to employment with the service.

### Theme 1: providing physical health behaviour coaching to participants with a mental health condition creates unique challenges and opportunities

#### Subtheme 1.1: the level of engagement with the service is perceived as different for participants with a mental health condition

Estimates of the proportion of participants of the service with a mental health condition varied greatly between coaches, ranging from 35 to75%. There was a prominent perception amongst coaches that participants may be reluctant to disclose a mental health condition in the early stages of coaching, but that such conditions (diagnosed or not) were disclosed or made apparent during later stages of the coaching program, when rapport was more developed.

*“It's common. Hard to put a number... Maybe about 35, 40%....Because if someone's got, say, bipolar, then they might not want to tell you that in the first, second call.... They need to build up that relationship… and I do think people, once they get started, they're quite open because we're not a face to them.”*(Coach 1)

Coaches perceived a number of core differences in program engagement between participants with a mental health condition compared to those who did not report a mental health condition. The former group was perceived as more likely to value contact with the service, particularly as a source of general social support.

*“I feel that some of the participants [with a mental health condition] I speak to value the communication as a contact. You know, they may not achieve weight-loss, for example, but they have a call that they can look forward to. And they have someone that cares and someone that's checking in with them…I think there's this assumption that perhaps they won't answer all the calls [but] I think often they will value the call more 'cause they may not necessarily have a lot of resources”* (Coach 1)

Coaches consistently expressed the feeling that the proactive service provision was particularly beneficial for engaging this participant group (i.e. the service contacting the participant for scheduled coaching calls rather than the participant being required to initiate contact). However, while some coaches expressed challenges around being a “sounding board” for this participant group, most talked about a greater level of engagement specifically brought on by the rapport built up through more in-depth conversations.

*“Sometimes they're even, they're more, I guess receptive to the coaching as well. I guess maybe because you do build that rapport with them, and so it's easy even for the coach to start having a conversation to be honest…sometimes I would presume that they just go into their other consults, and they're like, right, let's just talk about your mental health rather than the external factors.”* (Coach 2)

#### Subtheme 1.2: the complexity of achieving health behaviour change in consumers with a mental health condition

All coaches talked of a significantly more complex and difficult process involved in supporting achievement of health behaviour change goals in this group of participants. Achieving goals set together with the participant was compromised by a host of factors, with more prominent ones relating to prescribed medications inducing weight gain and directly hindering the capacity to lose weight, and others including dietary choices, motivation and self-efficacy;

*“More often than not, they're not really ready to change, they usually have to address a lot of other things before they make these changes…it's more contemplation. They understand that they probably should be doing this, but they feel that there's other barriers in the way.”* (Coach 2)

*“I also find that for a lot of people, the medication that they may be on as well, does…you know just has big impact on not only on their diet, but on their energy state as well to be able to get out there and exercise.”* (Coach 3)

The lower motivation among this participant group for attempting behaviour change in the immediate or short term, (as would align with the focus of the service), was one of the prominent features of the discussion with coaches. Although participants were perceived to overall express a desire to change their health behaviours and had taken that important first step to engage with the GHS, coaches indicated that they felt many in this group entered the program with a genuine interest and desire to move towards a healthier lifestyle, but were not yet ready or equipped to make behavioural changes. As a result, coaches reported their calls focussing on moving participants along from contemplating changes and talking about the outcomes they wished to achieve, rather than being ready and having the physical, mental and informational resources to take action. The content of calls was often around addressing participant attitudes towards making changes, and the confidence to do so;

*“The desire is there, but the actual willingness and drive to actually make a change can be hard. And we spend a lot of our conversations in that more sort of contemplative stage, where we're talking about, you know, "What benefits did you see?" You know, like doing the visualisation, getting them to actually picture themselves doing it, that sort of thing.”* (Coach 4)

*“I guess the struggles that play there is really, how we coach someone is getting them to see what changes they can make to exercise, and what changes they can make to nutrition, or how they can achieve weight loss. And a lot of the times, we are working with them to identify barriers, and one of those is often how they're feeling, they have good, bad... Good days, bad days.”* (Coach 5)

#### Subtheme 1.3: the relevance of different measures of success for people with a mental health condition

Due to specific and additional barriers experienced by this participant group, coaches explained that progress towards health behaviour change goals generally was much slower. Some coaches suggested that they often felt obliged to take a different approach to support and celebrate with the participant the often smaller changes made towards a larger health change journey.

*“So it might be recognising that perhaps it might be taking smaller steps for example, and I always do like to emphasise, you know, that step-by-step mentality...Rather than, you know, jumping big hurdles and making multiple changes all at once...'Cause recognising that it might be a bit more challenging for them.”* (Coach 4)

*“A lot of people aren't ready to sort of make any kind of changes. So it's just talking about... Maybe giving them a bit of knowledge about, okay, you know, even if they're thinking, "Right, okay, I'm thinking about going for a walk." You know, we might just go over just the benefits of doing that, and just talking through it, rather than actually setting a goal.”* (Coach 3)

Discussions with coaches alluded to ‘program success’ for participants with a mental health condition as being measured with a different yardstick. The measures of health behaviour change that were felt to be particularly beneficial to apply with this group centred around attitudes towards healthy eating or physical activity, or confidence and readiness to start making changes. Coaches reported that they used these different measures of success to assist participants in positively reflecting on their health journey.

*“But I find that often what success is to them might be a little bit different. Really just overcoming their I guess mindset to eating can be a huge... Which doesn't get reflected in the statistics, obviously.”* (Coach 5)

Coaches also noted however, that they often were privy to some specific positive impacts of coaching not currently captured in routine service data collection, such as participants reporting positive changes in mental health as a result of program engagement;

*“I always like to ask them what benefits they've noticed, and if they haven't really mentioned anything around, you know, their emotional well-being or mental health, I'll actually probe for that. I'll be like, "Have you noticed anything in how you've been feeling?" And then that could be a highlight for them, like, "Oh, actually yes, I've been feeling more positive." I actually had this one participant say to me, and I always remember this, he said that people have noticed that he's different, and I was like, "In what way?" And he was just like, "Just in my voice, I have a more positive voice now."* (Coach 4)

### Theme 2: the scope and characteristics of the service impact on the capacity to cater for participants with a mental health condition

#### Subtheme 2.1: service focus on physical health creates unique challenges for coaches and participants

Coaches acknowledged that whilst it was important for the coaching relationship and success of the program that participants talked openly about their mental health concerns, one of the consequences of this was that they, at times, felt ill-equipped to respond to disclosures of mental illness. Coaches perceived that participants reported problems that were outside the service’s focus on healthy lifestyle behaviour change, and that some provided information not directly related to physical health behaviour that may have been viewed as sensitive or personal in the context of service delivery. Some coaches alluded to participants with very acute problems not recognising the limited scope and focus of the service. Coaches talked of these being particularly challenging aspects of their work, and that further resources and training would be welcomed to enable them to provide appropriate support whilst staying within their scope of practice.

*“I often become amazed in the first call that I can have with someone, how much they tell me about so many things that have happened to them. Things like suicides, or people... Like things around them, and how they're feeling. And, I find that part rewarding… Someone to feel comfortable enough to tell me that. I guess what I struggle with then, is just the, "What do I do with that?”” (*Coach 5)

Participants expressing concerns relating to their mental health were referred to services such as Lifeline which could provide immediate support, and such services were considered valuable by coaches. In many cases, coaches also suggested that participants contact their general practitioner (GP) or mental health care worker if they were showing signs of needing support during a coaching call. However, some coaches talked of cases in which participants did not have a regular GP or mental health care worker to whom they could be referred. In these instances, coaches reported a sense of responsibility, yet feeling that they were unable to meet the needs of the participant.

*“It would be really great if we could perhaps link in with, like mental health services and be like, "Look, you know, perhaps it, it sounds like you need extra support, here's an organisation in your area that would be helpful. "I know that definitely does fall out of what our service does actually offer and provide but if it was possible, that would be really great ‘cause sometimes people aren't at that point where they can be talking to us, they definitely need support from like a mental healthcare worker…'Cause all we can really do is link back to their doctor. You know, checking with them, "Do you have a GP that you see? I'm thinking you should go and speak to your doctor about it."…but if they don't have a doctor, if they don't have a mental health care professional that they see...we can't be, then, linking them in with anyone or recommending anyone so that is where, that's where we might get a little bit stuck.”* (Coach 4)

#### Subtheme 2.2: working at the interface between health coaching and mental health requires specific training and resources

The experience of feeling they had a knowledge gap was expressed in some form by all coaches, and the majority identified a desire to develop more expertise in working with people with a mental health condition. This may reflect coach knowledge and experience in psychological constructs such as motivation, rather than knowledge and experience in mental illness per se. Coaches noted that as part of introductory training on commencing with the service, some information was provided around risk management (e.g., if a participant expressed suicidal ideations during a call), as well as appropriate referrals to mental health services. Whilst this training was perceived as beneficial and important, some coaches expressed an unmet need for more training around other areas pertaining to mental health; in particular, around the interface between mental and physical health, and how mental health may impact physical health behaviour change. Learning from other health professionals who had previous experiences in delivering care to mental health participants, which could be applied to their own service delivery, was one approach to upskilling suggested by several coaches.

*“I know we've had training on it before, but more of that like a refresher training, sort of maybe case studies or examples where people have had success with these kind of challenging participants or any that you've got to move from that contemplating to actually preparation, and action.”* (Coach 4)

Some coaches also indicated the significant value of having lived experience of a mental health condition in providing a better understanding of, and therefore ability to coach, those with mental health issues. A few spoke of the benefits of allowing coaches to gain a consumer perspective to enable a more empathetic appreciation of the challenges that mental health participants face.

*“I feel like it would be good to have, like a... I'm not sure exactly the right terminology, but like a… someone that's lived with, I guess, the extremes of being in that situation. And then, for them, you know, what sort of strategies would work?* (Coach 6)

Likewise, the potential for a psychologist or a mental health professional to be integrated as a part of the team was suggested to bridge the gap between addressing the mental and physical health of participants. On a practical level, coaches talked of several ways in which they could envisage the involvement of a mental health professional in the service. Firstly, as a member of the coaching team, providing direct support to participant with a mental health condition in achieving physical health behaviour changes. Secondly, as a resource for all coaches to consult with in regard to specific coaching questions. In this latter role, a readily accessible mental health professional was proposed as a means of debriefing from difficult conversations and managing the mental health of coaches themselves, as well as an educational resource for gaining insight into how to deal with certain scenarios with mental health participants. In either role, the inclusion of a mental health professional was seen as beneficial for coaches, allowing them to continuously learn and subsequently provide better care to their coaching participants.

*“Would be amazing if we could be having a psychologist as a health coach or even as, like, a go-to for us to be talking through challenging or difficult participants. But I think definitely even, you know, as a health coach on the service, it would be really great support.”* (Coach 4)

#### Subtheme 2.3: a consistent message from collaborating services

Coaches expressed the value of participants with a mental health condition receiving a consistent message around health behaviours from all health professionals involved in their care. This was discussed from the participants’ view point that they may be more likely to make positive health behaviour changes and see these as important when receiving the same advice from multiple sources;

***“****I do find that a lot of the time, you know, what I've talked about, then one of their other health carers have spoken about as well, and so they're getting that double message. It's like, "Oh, okay well yeah that person spoke about that and said, it would be really good for me", you know, for example going out for a walk every day, "and now you're telling me that as well." It's like, "Okay, well, maybe... Maybe if more than one person's telling me that, then maybe that's going to be working for me." So I do find that having, you know, a team works really well…that's when it starts making the job easy, when everyone else is on board saying the same thing to the person.”* (Coach 3)

***“*** *I think if health professionals are on the same page and not giving mixed messages, that's going to help reinforce to the patient that, "Yes, this is what I should be doing."”(Coach 1)*

For some coaches, the idea of being part of a ‘care team’ for a participant sounded ideal. This would allow for a two-way channel of communication to be established with other health professionals involved in a participant’s care which was felt to potentially facilitate more consistent, effective and holistic care.

*“I think it would be of benefit if there was more information being shared between health professionals associated with the patient...I'm not really sure of the ins and out, but...to have some input going both ways… or even have a three-way call. Even if it was for that first initial call where you're getting to know the patient.”* (Coach 1)

#### Subtheme 2.4: program parameters do not always fit the needs of participants with mental health issues

A number of program specific characteristics were identified that were perceived to impact engagement for participants with a mental health condition. Coaching programs currently do not include information or support specifically pertaining to mental health, and whilst most coaches had received some initial mental health training, it was not considered sufficient to fully address the impact of mental health on the capacity to make health behaviour change and enable them to provide optimum care. Depression was noted as a common mental health condition communicated by participants, and symptoms such as fatigue and low motivation were important factors that coaches felt ill-equipped to address;

“*I’d probably say that where the content is limited in this program is that support that we can really offer to overcome that number one barrier, which is their mental health.”* (Coach 5)

Balancing the organisational needs to complete a certain number of coaching calls in a shift whilst still meeting participant needs within the nominated call duration was noted as being the primary limitation of the service. Coaches expressed a desire to provide quality, empathetic care and behaviour change support, and that the confines of meeting targets governed by call length was a barrier to doing so. In particular, phone calls in which the focus involved managing a participant’s current mental health concerns inhibited coaches’ capacity to provide physical behaviour change support, and forced a prioritisation of mental over physical health by coaches, which it was perceived may impact the physical health outcomes these participants could achieve;

*“'Cause we have targets on how many calls we should be making a day, how many of them need to be successful. So a successful call is when you pretty much go through a whole call with someone where you are talking about... I guess it can be making a change, or it can be something that's impacting, so the barriers or motivations or whatever….that's the 15-minute call, which often they go overtime because the participant... So we have 20-minute time slots throughout the day, where we put our appointments in. Often, you don't even have that five minutes 'cause you're doing it in the call, and you're quickly wrapping, wrapping up, to get to the next one. So I find that's the biggest barrier.”* (Coach 5)

*“I find myself often running out of time with these calls and... 'cause yeah, it's just more complex sort of situation and like, if someone's distraught or someone's...you know, not in a good way, of course, you're going to spend that time and you won't sort of end the call. But, it makes it harder for you because you've got a full day of scheduled calls.”* (Coach 1)

One coach expressed that this was felt to be a major contributing factor in program attrition amongst this group of participants;

*“The time restriction of the 15 minutes, I know I've mentioned it before, but really taking the opportunity, especially in the first few calls, when you're trying to build rapport with someone, and then trying to allow them to speak about things, such as mental health, that's affecting them and get to learn how much that is impacting them. I don't think that the 15-minute time limit allows us to do that effectively… And that might be leading to the dropout rate.”* (Coach 5)

## Discussion

Despite the potential value of population-level telephone-delivered behaviour change coaching services to reduce the inequitable burden of preventable chronic disease experienced by people with a mental health condition, scant research has attempted to understand the delivery of such service provision for this group. To our knowledge, this is the first study to explore the delivery of telephone-delivered behaviour change support around healthy eating, physical activity and weight management to this population group from the perspective of coaches.

Service data collected at enrolment to the GHS indicates that participants with a mental health condition represent a considerable proportion of those enrolling (26%) [19]. It is interesting that coaches estimates were somewhat higher, one possible explanation being that some mental health concerns were revealed as coaching progressed. Coaches commented that the presence of a mental health condition was often mentioned once rapport was established between coach and participant during the course of coaching; suggesting that enrolment data may underestimate the proportion of participants who have a mental health condition.

The data identifies a number of opportunities and challenges for coach and participant. Telephone delivery as well as the proactive nature of the coaching program was viewed as particularly beneficial to participants with a mental health condition, with coaches suggesting that participants with such conditions were especially likely to value the contact. Evidence suggests that there is a correlation between mental illness and loneliness [37], and consistent evidence linking social isolation and loneliness to poor physical and mental health outcomes [38].Other population groups accessing this service have also expressed a valued experience of “having someone to talk to” [39]. The value of the contact provides an important opportunity for coaches to motivate participants to look forward to the next contact and remain engaged in the program.

Coaches perceived behaviour change to be more difficult for participants with a mental health condition, with additional factors sometimes including mental health symptoms and medications, as well as motivation and readiness to change health behaviours. These conclusions are consistent with literature reporting perceptions of both mental health consumers and health professionals from other settings [40–42]. For example, a survey of physical therapists working in mental health settings identified lack of motivation as the most frequently cited barrier to physical activity [40],while interviews with mental health participants identified medication side effects and symptoms relating to mental health as the most commonly reported barrier [42]. What may be perceived as motivation by health professionals or coaches is likely to be a more complex interplay of environmental, social, psychological and biological aspects of living with mental health conditions.

With these perceptions in mind, health coaches identified that for participants with a mental health condition the positive outcomes of service use may not be what would typically be captured in service evaluations. Currently, no quantitative data on behaviour change outcomes for participants with a mental health condition engaging with the GHS has been reported. However, coaches reported that often the coaching program can lead to a change in the attitudes and confidence towards creating and sustaining healthy behaviours – a necessary prelude for successful and sustainable behaviour change [43].Coaches also noted their perception of improvements in participant mental health following engagement with the service, irrespective of goal achievement. Systematic reviews have indicated that improvements in dietary intake [44] and physical activity [45] can lead to improvements in mental health. These gains can then be used in an iterative fashion to continue increasing motivation and confidence, albeit perhaps requiring a more lengthy engagement with the service to achieve health behaviour goals. The success of the GHS in delivering significant health improvements for those participants with a mental health condition may mean that further effort is required to increase completion of the coaching program, and encouraging participants to re-enrol to achieve their desired outcomes. A focus on strategies to retain participants to program completions [13] and encouraging re-enrolments [39] have been recommended in other GHS evaluation studies. Future service evaluations could also consider the inclusion of metrics for mental health.

Survey data from this study indicated that only 2 of 14 coaches had more than 12 months experience working with people with a mental health condition. As a means of both improving knowledge around mental health and providing more holistic care, coaches expressed a desire to be able to access and learn from practitioners with mental health expertise. Reports examining comorbidity and provision of physical health care among people with mental health conditions may be helpful in considering the possible shape of such training [46, 47]. Other options may include a mental health professional such as a psychologist being a member of the coaching team and supporting participants directly, or having such a professional as a resource for coaches, providing on-going education and opportunities for de-briefing. They also expressed value in learning from people who were living with a mental health condition, so as to enable them to be more empathic to the needs of this participant group. Also known as peerworkers [48], individuals with lived experience who support others dealing with a mental health condition provide a valuable source of knowledge in their ability to identify and communicate perspectives of both consumer and service provider, and have been previously explored as potential behaviour change delivery agents with promising results [49–52].

Coaches also reflected on the current structure of the service and its delivery which may impact the experience of participants with a mental health condition. Current coaching support to participants is limited to 15–20 min per call, with coaches reporting that targets to achieve a certain quantity of calls per day potentially hinders the provision of quality assistance and extra time for participants who may need it. Previous evaluations of the GHS have resulted in program changes and tailoring for specific groups, such as the development of an Aboriginal and Torres Strait Islander module [39]. Coaching participants who enrol in this module receive both culturally appropriate resources and an additional three coaching calls. Creation of the tailored coaching program has been noted to lead to improved participation and outcomes for Aboriginal participants [13, 39]. An additional strategy for increasing support for participants with a mental health condition may be collaboration with referring agencies. Currently, 45% of participants with a mental health condition engaging with this service are referred by a GP or other health professional [19]. Coaches indicated that greater communication between the service, participant and health professionals may also assist in providing consistent messages to the participant, which may impact management of biopsychosocial issues currently perceived as ‘lack of motivation’. Evidence from the Victorian Quitline, in which co-management includes doctors agreeing to manage comorbid health issues including mental health, suggests that this approach was acceptable to participants and associated with receipt of more Quitline calls and greater likelihood of having made a quit attempt at 2 months [53]. It may also be of value to consider a more collaborative approach with carers within the coaching process. An Australian study of carers of people with mental illness suggested an opportunity exists to build the capacity of carers to contribute to reducing the health risk behaviours among people with a mental illness [54].

A number of limitations should be noted in interpreting the study findings. The views presented are based on the experiences of health coaches in providing behaviour change support to people with a mental health condition, and the study does not purport to accurately reflect the consumers’ perspective. The sample size was small, and included only females from one telephone-service in NSW, Australia, and therefore the views expressed may not be generalizable to all coaches or more broadly to other behaviour change specialists such as psychologists across other settings and locations.

## Conclusion

Coaches of a telephone-based behaviour change support service perceived that participants with mental health conditions do make positive changes, most notably to their motivation or readiness to change behaviours (such as being more active or healthier eating) and that reduced mental health symptoms may also result. Coaches perceived that additional support for coaching staff may assist in improving their awareness of and confidence to address the specific barriers these participants experience. Previous qualitative reviews of the GHS have identified the value of tailoring for specific groups to increase participation and health behaviour outcomes [39]. The current findings suggest that similar adjustments for participants with a mental health condition may warrant consideration. Future studies on the views and experiences of participants, in addition to examining the nature and scale of outcomes achieved, would enable a more balanced understanding of the impact of a telephone health coaching service for those with a mental health condition.

## Data Availability

The datasets generated and analysed during the current study are not publicly available but are available from the corresponding author on reasonable request.

## Abbreviation

GHS: NSW Get Healthy Information and Coaching Service®
